# Achieving convenient CO_2_ electroreduction and photovoltage in tandem using potential-insensitive disordered Ag nanoparticles†
†Electronic supplementary information (ESI) available. See DOI: 10.1039/c8sc02576b


**DOI:** 10.1039/c8sc02576b

**Published:** 2018-07-20

**Authors:** Wanyu Deng, Lei Zhang, Hao Dong, Xiaoxia Chang, Tuo Wang, Jinlong Gong

**Affiliations:** a Key Laboratory for Green Chemical Technology of Ministry of Education , School of Chemical Engineering and Technology , Tianjin University , Collaborative Innovation Center of Chemical Science and Engineering , Tianjin 300072 , China . Email: jlgong@tju.edu.cn

## Abstract

Photovoltaic-electrochemical (PV-EC) systems can not only make full use of solar energy, but also transform CO_2_ into organic molecules. However, it is difficult to achieve PV-EC systems since most CO_2_ reduction catalysts are potential-dependent. This paper describes the rational design of potential-insensitive disordered Ag, which can achieve more than 90% faradaic efficiency (FE) for CO within a wide voltage range of 1.1 V in an electroreduction CO_2_ system. The system shows attractive activity under different photovoltage conditions in a PV-EC system. By employing *in situ* attenuated total reflection surface-enhanced infrared absorption spectroscopy (ATR-SEIRAS), we address the origin of the volcano peak of FE on Ag nanoclusters to understand the mechanism of the carbon dioxide reduction reaction (CO_2_RR). In addition, we find that the CO_2_RR on disordered Ag nanoparticles is a proton–electron coupling transfer (PECT) reaction mechanism, which may result in high activity in a wide potential range.

## Abstract 2

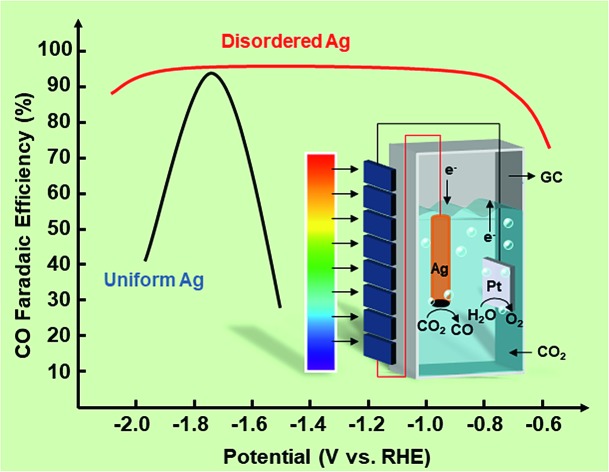
This paper describes the rational design of potential insensitive disordered Ag, which could achieve more than 90% faradaic efficiency for CO within a wide voltage range of ∼1.1 V in a photovoltaic-electrochemical systems for CO_2_ system.

CO_2_ in the atmosphere has recently reached the highest levels in human history due to the huge consumption of fossil fuels.^1^,^2^ Previous work showed that CO_2_ conversion to fuels and chemical feedstock could be achieved through electrochemical,^1^ photochemical,^3^ photoelectrochemical,^4^ thermochemical,^5^ and photovoltaic-electrochemical (PV-EC)^6^–^9^ routes. Among these approaches, the PV-EC system has several advantages, such as potential large-scale amplification due to the modular electrolyzer design and ease of integration with solar energy sources. Therefore, the utilization of electrical energy generated by photovoltage to drive the electrochemical CO_2_RR to yield fuel and chemical feedstock is feasible and of great prospect.^6^–^9^ However, it has been reported that the distribution of CO_2_RR products is potential-dependent.^10^–^13^ Hence, CO_2_RR coupled components need to be carefully selected. Meanwhile, under actual operation conditions, the fickle change of sunlight intensity can also affect the photogenerated voltage. Therefore, it is important and necessary to design a potential-insensitive catalyst with high efficiency and selectivity.

To achieve a PV-EC system, selection of the catalyst is also a key factor since the activity and selectivity of the electrocatalyst have a great effect on system efficiency and product distribution. Among various types of electrocatalysts, Au, Ag, Zn, and Pd are known to produce carbon monoxide (CO) with varying faradic efficiency, which depend on the potential.^10^,^14^–^16^ Meanwhile, Au and Ag based catalysts exhibit the highest activity and selectivity towards CO during the electrocatalytic CO_2_ reduction process. Since Ag is much more abundant and less expensive than Au, it is considered as the more promising electrocatalyst for large-scale production of CO. Hwang and co-workers investigated the relationship between the particle size of Ag nanoparticles and CO_2_RR activity.^10^ Subsequently, Luo and co-workers combined experimental and computational efforts to propose that CO_2_RR activity and selectivity on Ag could be greatly promoted by creating optimal facets and edge sites.^17^ However, almost all the Ag catalysts showed an apparent volcano peak with potential during the CO_2_RR process, and the potential window for optimal activity is too narrow for a PV-EC system, which limits the large-scale utilization of catalysts in the solar-driven electrochemical CO_2_RR.

This paper describes the discovery of disordered Ag nanoparticles on a carbon support catalyst, which could achieve 90% FE for CO in a wide voltage range instead of showing an apparent volcano peak. In order to test the superiority of the potential-insensitive catalyst, a new PV-EC system was fabricated firstly with the disordered Ag catalyst, Pt foil and a six-section a-Si solar cell as the cathode, anode and photovoltage source, respectively. It was found that the disordered Ag nanoparticles showed attractive and potential-insensitive activity. Finally, by using ATR-SEIRAS spectra, the CO_2_RR mechanism and the relationship between CO FE and potential over Ag nanoparticles were explained.

A series of carbon-supported Ag nanoparticles were prepared. The disordered particle size Ag was controlled by adjusting the stirring speed and heating rate. Meanwhile, the uniform particle diameter Ag was synthesized by adjusting the reaction time and Ag precursor species (see the ESI† for experimental details).^10^Fig. 1a shows that the disordered Ag size distribution is irregular, while uniform Ag (Fig. 1b–d) size distributions are 3.2 ± 0.05 nm, 4.9 ± 0.04 nm, and 11.0 ± 0.1 nm, respectively. The *d*-spacing of all the Ag nanoparticles is 0.23 nm which corresponds to the Ag (111) crystal plane (Fig. 1e–h). It can also be found that these nanoparticles have a face-centered cubic Ag crystal structure from the X-ray diffraction (XRD) data in Fig. S1.†

**Fig. 1:**
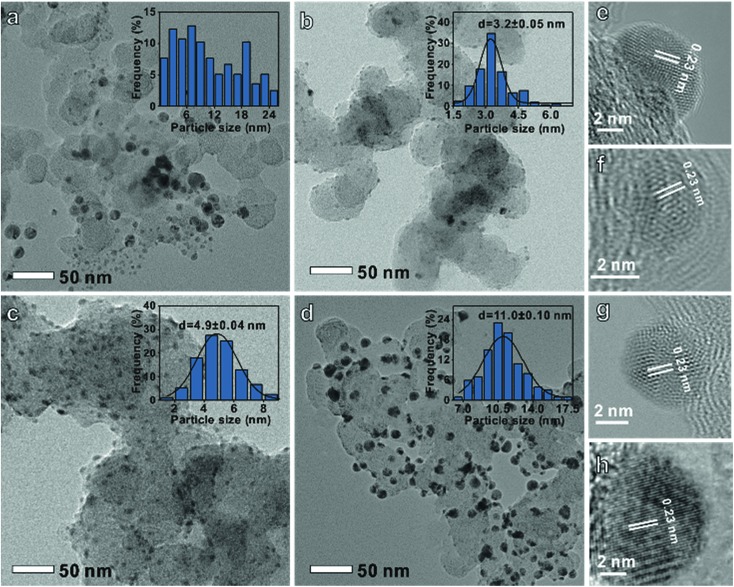
Transmission electron microscopy images and histograms of particle-size distribution of (a and e) disordered, (b and f) 3 nm, (c and g) 5 nm and (d and h) 11 nm Ag on a carbon support.

To test the CO_2_RR performances on Ag nanoparticles, controlled potential electrolysis of CO_2_ was performed in a three-electrode system (Fig. S2a†). Under the test conditions, CO FE of uniform Ag nanoparticles and Ag foil shows a volcano peak with potential dependence (Fig. 2a) in 0.1 M KHCO_3_, wherein the HER is facile at low overpotentials and CO_2_RR is prone to occur with increased potential, while CO FE decreases at higher potentials. On 3 nm, 5 nm and 11 nm Ag, maximum efficiencies of 83%, 90% and 95% are achieved at –1.1, –0.6 and –1.4 V, respectively. Interestingly, CO FE of disordered Ag is more than 90% from –0.6 V to –1.7 V. The highest CO FE is close to 70% at –1.3 V over Ag foil, which matches well with the previous work.^18^ To rule out the influence of the electrolyte, we tested CO_2_RR in 0.1 M KCl (Fig. 2b). The activity of Ag catalysts is not affected by KCl electrolyte. In contrast, the performance is slightly better because of the Cl^–^ adsorbed on the electrode surface inhibiting the HER.^19^,^20^

**Fig. 2:**
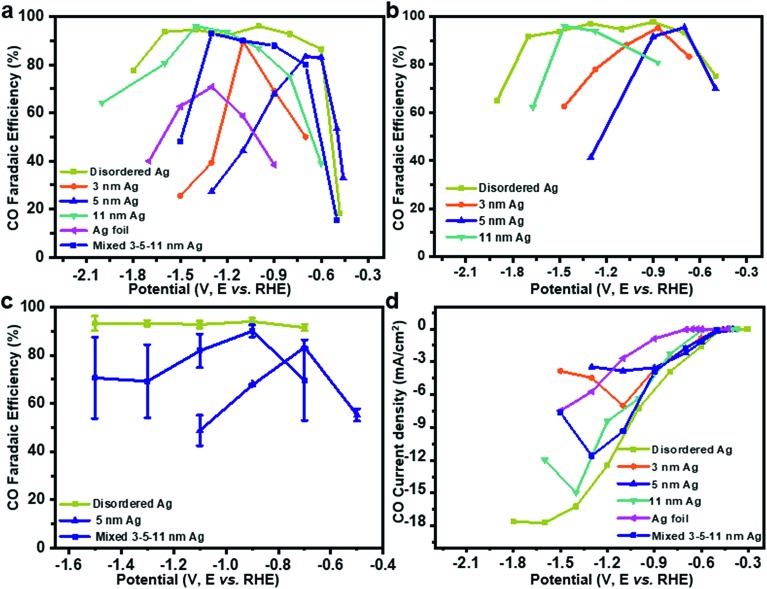
CO faradaic efficiency depending on applied potential in CO_2_ saturated (a) 0.1 M KHCO_3_ and (b) 0.1 M KCl, (c) CO faradaic efficiency on a simple mixture of 3–5–11 nm, disordered and 5 nm Ag in 0.1 M KHCO_3_ with error bars. (d) CO partial current density depending on applied potential.

It should be noted that achieving such a wide voltage range of CO_2_RR activity is difficult, even by mixing 3, 5, 11 nm Ag in the mass ratio of 1 : 1 : 1. It might be attributed to the uneven distribution of Ag particles on the glassy carbon electrode surface. When the same ink was tested five times (Fig. 2c), a simple mixture of 3–5–11 nm Ag showed a much larger error bar than disordered Ag or 5 nm Ag, which proved that the performance of mixed 3–5–11 nm Ag exposed to the glassy carbon electrode surface is different for the five times. Therefore, it is hard to achieve potential-insensitive activity by mechanically mixing different particle size Ag nanoparticles.

The current density for CO increases with potential on disordered Ag (Fig. 2d), indicating accelerated reaction rate for CO_2_ reduction and it is maintained at –16.7 mA cm^–2^, which is probably caused by the limited mass transport of CO_2_ in 0.1 M KHCO_3_ solution.^21^,^22^ However, the current density of uniform Ag decreases at a higher potential. A durability test was performed with Ag foil and disordered Ag for five hours at –1.1 V (the best potential for CO_2_RR on Ag foil) and –0.7 V, respectively. Although the current density is stable for both Ag foil and disordered Ag during the 5 hour test, the decline of FE for CO on disordered Ag is 16.0% compared to 54.3% on Ag foil (Fig. S3a and b†). It shows that disordered Ag is much more stable than Ag foil.^10^ From the TEM (Fig. S4†) and X-ray photoelectron spectra (XPS, Fig. S5†) after 5 hours CO_2_RR, the overall morphology of Ag nanoparticles remains unchanged and the Ag 3d peak is basically the same. To compare with previous electrocatalysts for the CO_2_ reduction to CO, the activity data are summarized in Table S1 and Fig. S6.† Disordered Ag shows a very competitive FE for CO as well as the widest voltage range at the FE higher than 90%.

Further, solar-driven CO_2_ reduction was performed in an H-cell filled with 0.1 M KHCO_3_ solution (Fig. S2b†). Before the PV-EC system test, it is necessary to determine how much light induced voltage is needed to drive both CO_2_RR and oxygen evolution reaction (OER). The linear sweep voltammetry measurements were conducted on disordered Ag and Pt foil in 0.1 M KHCO_3_. As deduced from Fig. 3a, a voltage of 2.4 V was required. At the same time, a six-section a-Si with an area of 25 cm^2^ can provide an open circuit voltage of 3.38 V and a short circuit current of 4.6 mA cm^–2^, which can meet the demand of the CO_2_RR and photovoltage in tandem (Fig. 3b). During the PV-EC system test, a potential of –0.75 V can be observed at the cathode (Fig. 3c). Through product analysis it was found that the disordered Ag exhibits the highest CO FE of 92.7%, while 3 nm, 5 nm and 11 nm Ag can only achieve 48.0%, 87.2% and 68.2%, respectively (Fig. 3d), which is consistent with the previous measurement results at –0.75 V in a three-electrode system (Fig. 2a). It should be noted that the selection of photovoltage was limited to the cathode potential. To further illustrate that the potential-insensitive disordered Ag is the best choice for the tandem cell, a ten-section random sheltered a-Si solar cell is used. Fig. S7a† shows that the cathode potential is about –1.0 V using a ten-section a-Si solar cell. The disordered Ag exhibits the best activity of 91.8% and the FE for CO of 3 nm, 5 nm and 11 nm Ag are 60.1%, 45.1% and 74.3% (Fig. S7b†), respectively. Even when the area of the twelve-section a-Si solar cell changes randomly (Fig. S7c†), the disordered Ag still exhibits the best activity of 92.8% and the FE for CO of 3 nm, 5 nm and 11 nm Ag turn into 79.9%, 71.3% and 25.3% (Fig. S7d†), respectively. In the PV-EC system, it is difficult for the output voltage of practical solar cells under sunlight to remain stable and achieve a specific cathode potential. Fortunately, the disordered Ag is potential-insensitive and thus CO FE will not be affected with changing potential. The durability test with disordered Ag in the PV-EC system was also conducted, in which the decline of FE for CO was close to that of the three electrode system (16.8%) for five hours (Fig. S3c†). It can be speculated that enhancement in the stability of the PV-EC system could be achieved by improving the stability of the cathode catalyst. At the same time, the decline of FE for CO on 3 nm, 5 nm, 11 nm Ag nanoparticles is almost the same as in the case of uniform Ag catalysts (Fig. S3d–f†), which could be determined by the catalyst preparation method. Due to the low short circuit current (4.6 mA cm^–2^) of the commercially purchased a-Si solar cell caused by excessive series, the solar to cathode product (STC) efficiency of our PV-EC system is 0.1% (see the ESI† for further detailed calculations).

**Fig. 3:**
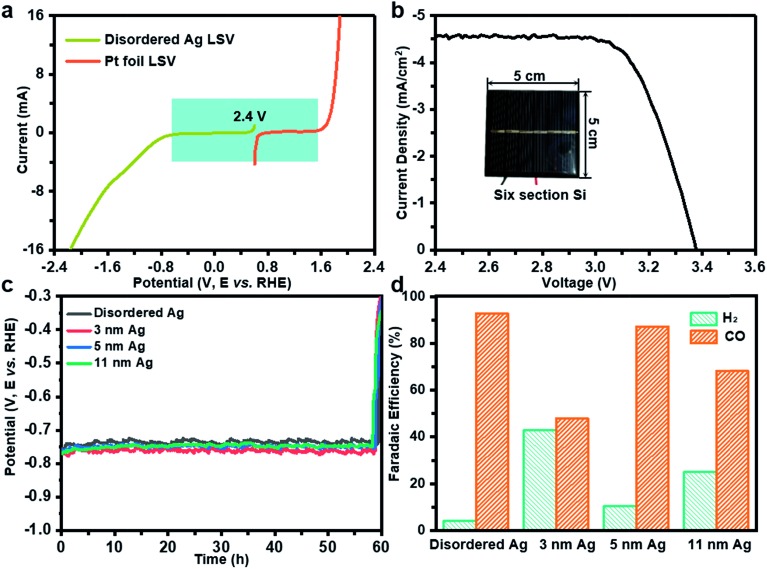
(a) Performance of the disordered Ag and Pt film towards the CO_2_RR and OER by linear sweep voltammetry at a scan rate of 50 mV s^–1^ in 0.1 M KHCO_3_. (b) The current density–voltage characteristics of a six-section a-Si solar cell were measured under AM 1.5G illumination. (c) Cathode voltage and (d) CO FE in the PV-EC system driven by a six-section a-Si solar cell.

From a thermodynamic perspective, the free energy diagram (Fig. S8†) indicates the competitive relationships between the CO_2_RR and HER on the Ag surface at different potentials.^17^,^23^ When a potential of 0 V is applied (purple line), the formation of *COOH for the CO_2_RR and that of *H for the HER are still uphill energy barriers, which are about –1.2 eV and –0.8 eV, respectively. At this stage, neither the CO_2_RR nor the HER can happen. With the increase of potential to –0.8 V (green line), the energy barrier for the HER can be overcome, but it is still not achieved for the CO_2_RR. As a result, the HER takes place and causes low CO FE at low potential. When the energy barrier for the CO_2_RR is overcome (–1.2 V, red line), the CO_2_RR starts to occur and CO FE increases (Fig. 2a). When the potential exceeds a specific value, the CO_2_RR dominates. For example, at –1.6 V (blue line), a much lower energy barrier of the CO_2_RR is obtained compared with the HER. From a dynamic point of view, the Tafel slopes of disordered or uniform Ag and Ag foil are close to 128 mV dec^–1^ (Fig. S9†), which indicates that one electron transfer is the rate-determining step for these catalysts.^24^ It is worth noting that the two electron transfer processes are involved in the formation of *CO for the CO_2_RR, while only one electron is needed for the HER. Even if it requires two *H to form H_2_, it only takes one electron to form *H. Hence, the CO_2_RR is more sensitive to potential, because the potential energy of electrons changes with cathode potential. For example, if two electrons are required for an elementary reaction step, the barrier of the reaction increases by –2 eV when the potential of –1 V is increased. Thus, more electron transfer steps would be more sensitive to potentials. By rights, CO FE and partial density current should increase with potential until mass transfer control is achieved. However, the uniform Ag catalyst shows a volcano peak of CO FE (Fig. 2a), which may suggest a change in mechanism with potential. It is noteworthy that the Tafel slope can only suggest the reaction process under low potential.

To understand the CO_2_ reduction reaction process on Ag nanoparticles, the ATR-SEIRAS spectra were used to observe the intermediate process (see the ESI† for experimental details). According to Fig. 4a, the peak at 1650 cm^–1^ is the overlays of H–O–H bending and C=O asymmetric stretching (1660 cm^–1^) of *COOH.^25^–^27^ The peak at 1378 cm^–1^ belongs to symmetric *COO^–^ stretching and C–O stretching.^28^ Other absorptions appearing at 1290 cm^–1^ can be ascribed to the C–OH stretch of *COOH.^25^,^26^ Therefore, the formation of the *COOH intermediate from 1378 cm^–1^ and 1290 cm^–1^ and the formation of *COO^–^ intermediate by comparing their peak strengths can be extrapolated. The detail band assignments are shown in Table S2.† To rule out the influence of the Au substrate and Ag–C catalyst itself, the Au substrate was recorded in CO_2_ saturated 0.1 KCl electrolyte and the disordered Ag catalyst was recorded in Ar saturated 0.1 KCl electrolyte, respectively. No absorbance was observed from 1400–1270 cm^–1^ at different applied potential (Fig. S10†).

**Fig. 4:**
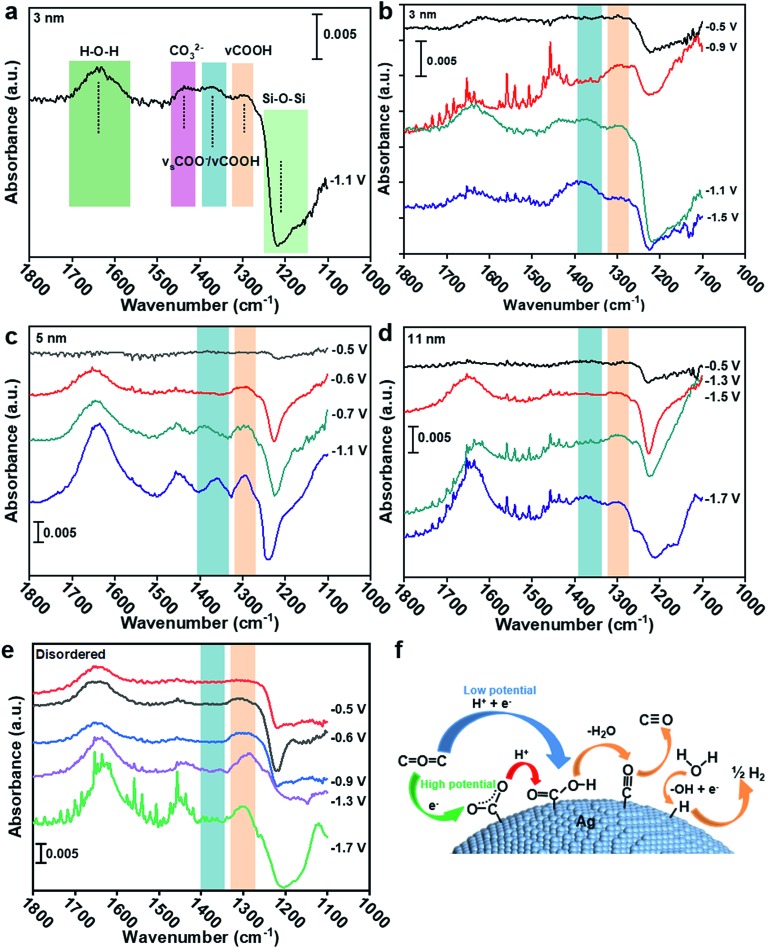
(a) ATR-SEIRAS spectra of 3 nm Ag in CO_2_ saturated 0.1 M KCl electrolyte at –1.1 V. ATR-SEIRAS spectra of (b) 3 nm, (c) 5 nm, (d) 11 nm and (e) disordered Ag in CO_2_ saturated 0.1 M KCl electrolyte at different potentials. (f) Reaction mechanism for the CO_2_RR and HER on the Ag surface.

In Fig. 4b, the ATR-SEIRAS spectra were recorded on 3 nm Ag at –0.5, –0.9, –1.1 and –1.5 V. The clear difference in the spectrum is that the 1378 cm^–1^ peak is larger than the 1290 cm^–1^ peak at –1.1 V and –1.5 V, indicating the coupling of C–O stretch of *COOH and the symmetric stretch of *COO^–^. However, the 1378 cm^–1^ peak is hard to be observed at –0.5 V and –0.9 V, despite the obvious occurrence of the 1290 cm^–1^ peak at –0.9 V, implying no sign of the *COO^–^ intermediate. It can be indicated that the CO_2_RR on 3 nm Ag is a proton–electron coupling transfer (PECT) process at low potential (<–0.9 V, Fig. 4f). However, at high potential (>–1.1 V), the reaction mechanism changes, in which an electron first transfers to form *COO^–^ and then a proton is introduced to form *COOH (Fig. 4f),^28^ which is a rate-limiting step for the CO_2_RR.^23^ Since the rate-limiting step is not electron transfer, the CO_2_RR would not be affected by potential. Therefore, CO FE begins to decrease and shows a volcano peak. The decrease of CO current density (Fig. 2d) is because of the competition between the CO_2_RR and HER; as more protons participate in the HER process, it will be difficult for *COO^–^ to accept protons generating *COOH. For 5 nm and 11 nm Ag (Fig. 4c and d), *COO^–^ can only be found at potentials higher than –0.7 V and –1.5 V, respectively. Hence, the trigger potentials for 3 nm, 5 nm and 11 nm Ag are –1.1 V, –0.7 V and –1.5 V, respectively, which are very close to the potentials of the best CO FE –1.1 V, –0.6 V and –1.4 V (Fig. 2a). For disordered Ag, the peak at 1378 cm^–1^ is less obvious than that at 1290 cm^–1^ at five tested potentials, indicating no sign of the *COO^–^ intermediate or a trigger potential from –0.5 V to –1.7 V (Fig. 4e). It shows that the CO_2_RR on disordered Ag is a PECT process (Fig. 4f), where the CO_2_RR can be promoted with the increase of potential against the HER. As a result, there is no volcano peak of CO FE on disordered Ag.

In summary, disordered Ag catalysts with considerably high activity in a wide voltage range instead of showing a volcano peak were successfully synthesized. From the solar-driven CO_2_ reduction experiment, the disordered Ag can achieve efficient CO_2_ reduction under different photovoltage conditions. By using ATR-SEIRAS, it can be found that disordered Ag undergoes the PECT process with increasing potentials. By contrast, the PECT process on uniform Ag changes to an electron–proton separated transfer process at high potential. In a wider PV-EC system context, the rational design of the potential-insensitive Ag catalyst for PV-EC CO_2_ reduction can inspire the design of other potential-insensitive CO_2_RR electrocatalysts for production of HCOOH, CH_4_, CH_3_OH, *etc.*

## Conflicts of interest

There are no conflicts to declare.

## Supplementary Material

Supplementary information
