# Supplementary material for: Achieving convenient CO2 electroreduction and photovoltage in tandem using potential-insensitive disordered Ag nanoparticles
Source: Chem Sci. 2018 Jul 20;9(32):6599–604. doi: 10.1039/c8sc02576b (PMC6115632; doi:10.1039/c8sc02576b)
Supplement: Supplementary file 1 [file SC-009-C8SC02576B-s001.pdf]

Electronic Supplementary Material (ESI) for Chemical Science.
This journal is © The Royal Society of Chemistry 2018

Supporting Information

Achieving Convenient Tandem of CO_2 Electroreduction and Photovoltage Using Potential-Independent Disordered Ag Nanoparticles

Wanyu Deng[1], Lei Zhang[1], Hao Dong, Xiaoxia Chang, Tuo Wang, and Jinlong Gong*

Key Laboratory for Green Chemical Technology of Ministry of Education, School of Chemical Engineering and Technology, Tianjin University; Collaborative Innovation Center of Chemical Science and Engineering, Tianjin 300072, China

*Corresponding author: jlgong@tju.edu.cn

1 These two authors contributed equally to this work.

Experimental Procedures

Chemicals and Materials

Silver nitrate ($AgNO_3$, 99.99%) was purchased from Shanghai Aladdin Biotechnology Co., Ltd. Silver acetylacetonate (Ag(acac)) was purchased from Alfa Aesar. Cysteine (>98.0%) was purchased from Tokyo Chemical Industry Co., Ltd. Ethylene glycol (EG, AR) was purchased from Tianjin Yuanli chemical Co., Ltd. 1-octadecene (ODE, 90%) was purchased from J&K Chemical. Activated Carbon was purchased from Tianjin Chilong Water purification material Technology Co., Ltd. Ethanol (99.8%) was purchased from Tianjin Yuanli chemical Co., Ltd. Hydrogen nitrate (AR) was purchased from Yongqing County Yongfei Chemical Reagent Co., Ltd. Sodium hydroxide (NaOH, ≥98%) was purchased from Alfa Aesar. Potassium bicarbonate ($KHCO_3$, 99.5%) was purchased from McLean reagent co., Ltd. All the reagents were used without any purification process. High purity water (18.25 MΩ·cm) supplied by a UP Water Purification System was used in the whole experimental processes. Six-section a-Si solar cell (5×5 cm^2) was purchased from Children's Dream Science and Technology Museum DIY Module Accessories on Tao bao.

Characterization

The morphology and particle size of the Ag were characterized by transmission electron microscopy (TEM, JEM-2100). The average particle size and crystal structure was determined by X-ray diffractometer (D/MAX-2500) with Cu Kα radiation (λ = 1.5416 Å) at 40 kV and 140 mA and X-ray photoelectron spectra (XPS, EscaLab MK). XRD spectra was collected over a 2θ range of 20-80° at a scanning speed of 8° per minute.

Methods

Different particle size Ag nanoparticles on carbon support were fabricated according to the previous references with a little adjustment.[10] In brief, activated carbon was refluxed for 3 hours at 80 °C in hydrogen nitrate with a volume concentration of 10%. For pH=7 carbon

support, the pH of activated carbon was adjusted by 10% NaOH. In a standard synthesis process of disordered Ag, 10 mL of EG, 0.002 g of cysteine, and 0.02 g of prepared activated carbon were sonicated for one hours. 0.02 g of $AgNO_3$ was dissolved in 10 ml of EG without stirring and this solution was heated to 50 °C in 5 min and last for 20 min with stirring. Then the prepared carbon solution was transferred to the silver precursor solution at 50 °C, and the mixed solution was kept at 50 °C for 10 min prior to heating to 160 °C in 30 min and last for 3 hours. When the solution cooled to room temperature naturally, the final samples were collected by centrifugation, washed several times with ethanol. For 3 nm and 5 nm Ag were synthesized by adding 0.02 g of $AgNO_3$ to 10 mL of EG with vigorous stirring and slowly heating to 50 °C for 20 min. The other experimental conditions were just like disordered Ag, only for one and three hours at 160 °C, respectively. For 11 nm Ag, 20 mg of Ag(acac) was dissolved in 10 mL ODE under nitrogen flow with vigorous stirring and 10 mL of ODE, 0.002g of cysteine, and 0.02g of prepared activated carbon were sonicated for one hours. The procedure was the same as the method above only for three hours at 200 °C. For distinction, the disordered particle size Ag nanoparticles were called disordered Ag and the average size of uniform particle diameter Ag in each sample was used in the sample name, for instance, 3 nm Ag indicated that Ag nanoparticles in the Ag catalyst had an average particle size of 3 nm.

Electrochemical Test

Electrochemical measurements were carried out in a homemade H-type cell with a cathodic and anodic compartment separated by a Nafion membrane, using a three-electrode configuration, with the glassy carbon electrode as WE, a platinum foil ($2 \times 2\ cm^2$) as CE, and saturated Ag/AgCl as the RE. A glassy carbon electrode with a diameter of 8 mm was used on which 40 μL of the ink formulation solution (catalysts 20mg were dissolved in the disordered 2 mL of ethanol and 20 μL of 2 vol% Nafion solution) was dropped. An

electrochemical workstation (IVIUM CompactStat.e20250, Netherlands) was used to measure the current and provide voltages. A CO_2 saturated 0.1 M potassium bicarbonate ($KHCO_3$) solution (pH 6.8) were used as the electrolyte. In order to achieve solar-driven CO_2RR cell (Figure S2), the glassy carbon electrode as cathode, platinum foil as anode and a six-section a-Si solar cell (5×5 cm^2) as driving force. The simulated solar illumination was obtained by passing light from a 300 W Xenon arc lamp (Beijing Perfectlight Technilogy Co. Ltd., Microsolar 300 UV) equipped with an AM 1.5 filter, and the power intensity of the incident light was calibrated to 100 mW/cm^2 at the surface of solar cell. At the same time, in order to detect the potential of cathode, a saturated Ag/AgCl was used as RE and two electrochemical workstations were used to measure the current and the potential of cathode, respectively. Before the reaction, the cathode and anode compartment were filled with 70 mL and 80 mL of the electrolyte, respectively. The cathode was connected to a gas circulation system with a ten-port value (VICI) for on-line sampling to a gas chromatograph (Ruimin GC 2060, Shanghai). The gas circulation system was primarily made of stainless steel tubing and a home-made gas pump for the circulation of the gas. A mechanical pump was connected into the system to exhaust the carrier gas of the gas chromatograph when switch back the ten-port value. A pressure gauge was also connected into the system to monitor the gas pressure. The total volume of the gas in the circulation system after filling the reactor with electrolyte was 150 mL. Before the start of the reaction, both the electrolyte and circulation system were purged with CO_2 (≥99.995%) for 30 minutes to achieve CO_2 saturation and remove air. Then the system was sealed and the initial CO_2 pressure was kept atmospheric. There was no more CO_2 purged into the closed system during the reaction. The gases in the closed circulation system were continuously circulated through the electrolyte for the entire reaction period. All the potentials vs. Ag/AgCl were converted to the reversible hydrogen electrode (RHE) using E (*vs*. RHE) = E (*vs.* Ag/AgCl) + 0.197 V + 0.0591 × pH.

Solar to Cathode Product Conversion (STC) Efficiency Calculation[8]

Considering the CO_2RR current of 1.5 mA (Figure S3), corresponds to an electric charge of 1.5 mC ($9.36 * 10^{15}$ electrons for one second). These charges are used to produce H_2 and CO which both need two electrons to be formed. Over PV-EC system driven by a six-section a-Si solar cell, an average faradaic efficiency of 90.1% for the CO production and 9.7% for the H_2 production were measured during one hour. Thus, we can calculate that:

$0.901 * 9.36 * 10^{15} / 2 = 4.22 * 10^{15}$ molecules of CO ($7.01 * 10^{-9}$ moles)

$0.097 * 9.36 * 10^{15} / 2 = 4.54 * 10^{14}$ molecules of H_2 ($7.57 * 10^{-10}$ moles).

Considering the respective molar masses, we obtain 0.196 μg of CO and 0.0015 μg of H_2. Taking into account the lower heat value and higher heat value for CO and H_2 of 283.5 kJ/mol and 286 kJ/mol, respectively, the specific power of the produced products can be obtained as 10125 kW/kg for CO and 143330 kW/kg for H_2.

Therefore, η_{STC} is given by

$$\eta_{STC} = (0.196\ \mu g\ CO \times 10125\ kW/kg + 0.0015\ \mu g\ H_2 \times 143330\ kW/kg)\ /\ total\ integrated\ power\ input$$

where the input power (for 25 cm^2 illuminated solar cell area) is the incident light intensity (2500 mW). Thus, the solar to cathode products efficiency of our device was 0.09 %, which was smaller than most reported work[8-9] owing to excessive resistance and low current density caused by multiple batteries in series.

ATR-SEIRAS Test

Au film deposition. According to Miyake method, Au film electrodes were deposited directly on a Si prism used in ATR-SEIRAS.[S1] To get a flat reflector, the Si crystal was polished with a 0.05 μm Al_2O_3 polishing powder. Then, in order to clean the organic contaminants on crystal, the Si crystal was immersed in Piranha solution (a 3:1 by volume

solution H_2SO_4 and H_2O_2) for 1 hour. Before deposition, in order to improve adhesion of the Au film, we immersed the reflecting plane of the crystal in 40% NH_4F solution for 90s to remove the oxide layer and generate a hydrogen-terminated surface.[S2] The reflecting plane of Si crystal was then immersed in a mixture of 68 μL of 40% HF and 4 mL of Au plating solution preparing by constant volume 0.1050 g NaOH and 0.2276 g $HAuCl_4$, 0.1337 g NH_4Cl, 0.98453 g Na_2SO_3, 0.6205 g and $Na_2S_2O_3 \cdot 5H_2O$ to 100 mL for 5 min at 55 °C. Different Ag electrodes were made by droping 100μL of the ink formulation solution (catalysts 20 mg were dissolved in the disordered 2 mL of ethanol and 20 of 2 vol% Nafion solution) on Si prism with Au film. All reagents were purchased from Alfa Aesar without further purifying.

In ATR-SEIRAS experiments. The Si prism with incidence of a 60° angle, Pt foil and an Ag/AgCl electrode were served as the working electrode, counter electrode and reference electrode. A H-type electrochemical cell, separated by Nafion membrane, was designed to accommodate the Si prism and to avoid any possible cross-contamination from the counter electrode. A Nicolet iS50 FT-IR spectrometer equipped with MCT detector and a Pike Technologies VeeMAX Ⅲ ATR accessory were employed for the electrochemical ATR-SEIRAS. Before performing experiment, 5 L/min N_2 was used to purge the optical path system for one hour to reduce the influence of CO_2 and H_2O(g) in air. All spectra were collected with a 4 cm^{-1} resolution and 8 scans. The electrolyte used was 0.1 M KCl due to its invisibility for infrared radiation, which was purged with CO_2 for 30 min (pH=3.94) before test. All the back ground was collected without applied potential in CO_2 saturated 0.1 M KCl and spectrum was collected with a fixed potential at 10 min. There will exist an inverted peak because of SiO_2 reduction at cathod.[S3]

Results and Discussion

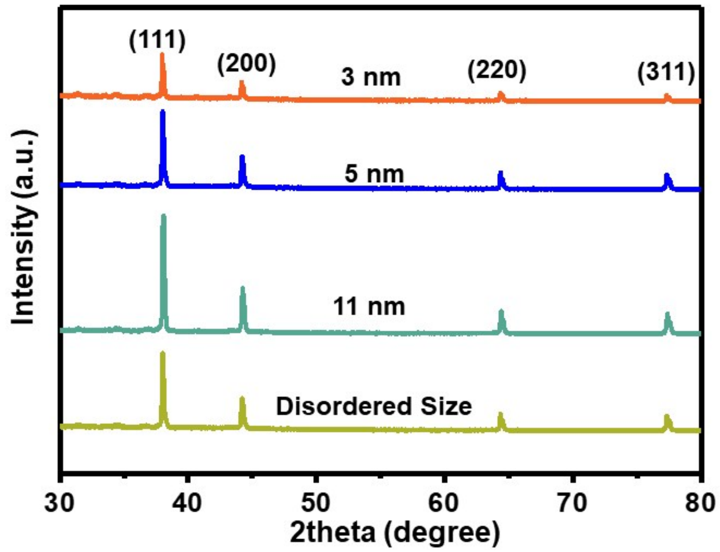

Figure S1. XRD data of 3 nm, 5 nm, 11 nm and disordered Ag with only crystalline face centered cubic Ag peaks present.

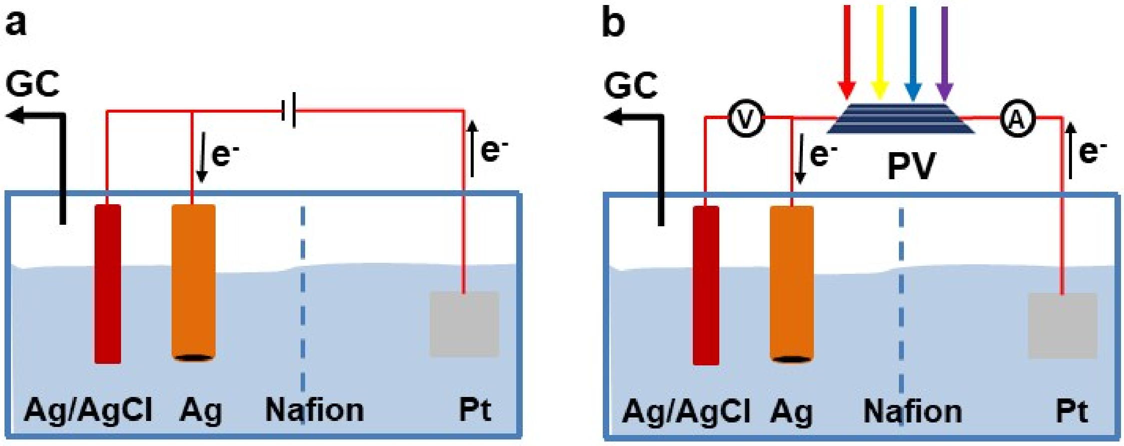

FigureS2. Schematic representation of (a) three-electrode system (b) PV-EC system.

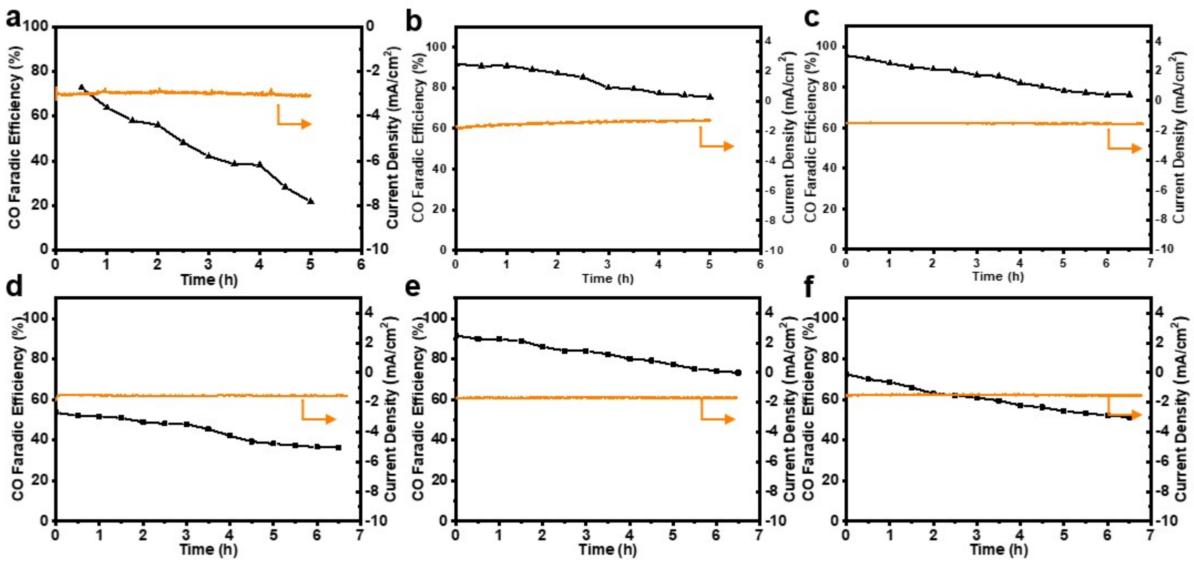

Figure S3. Durability test with (a) Ag foil at -1.1 V, (b) disordered Ag sample at -0.7 V and (c) disordered, (d) 3nm, (e) 5nm, (f) 11nm Ag nanoparticles in PV-EC system driven by a six-section a-Si solar cell. Durability test was performed in 0.1 M $KHCO_3$.

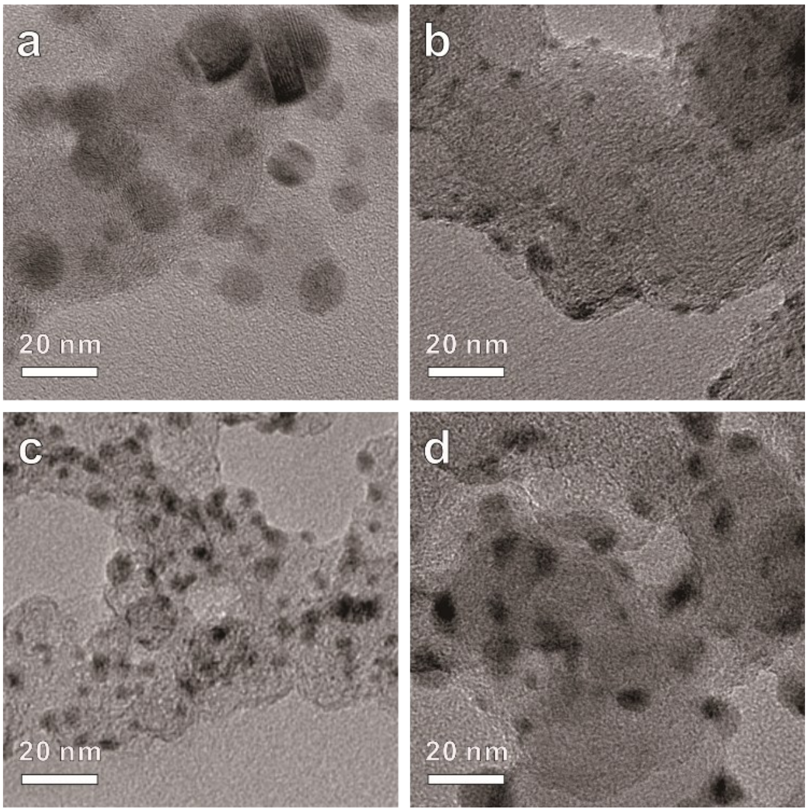

Figure S4. TEM images of (a) disordered, (b) 3 nm, (c) 5 nm and (d) 11 nm Ag nanoparticles after CO_2RR in -1.2 V *vs.* RHE.

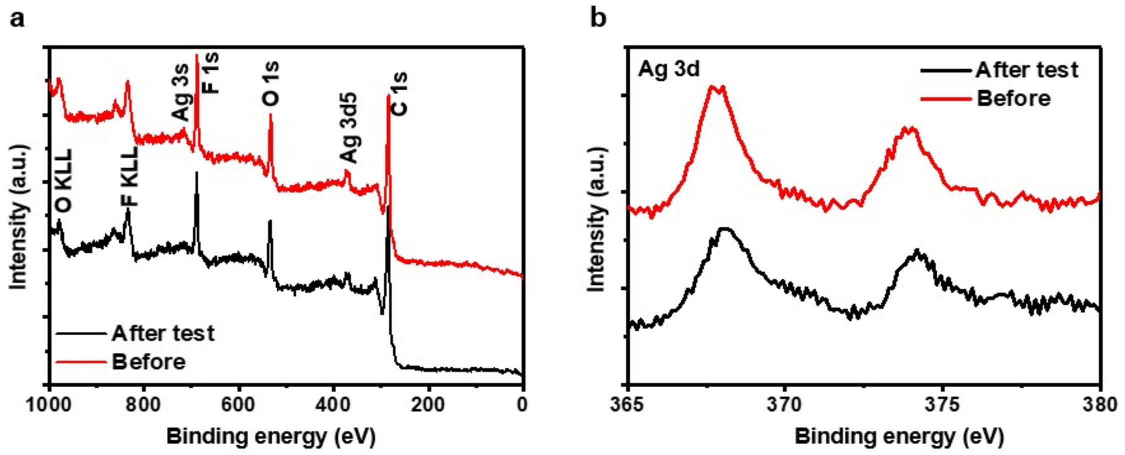

Figure S5. XPS images of disordered Ag before and after reaction in -1.2 V *vs.* RHE.

Table S1. Comparison of different metal electrocatalysts for CO_2 reduction to CO.

Sample	Electrolyte	CO FE range (%)	Voltage range of CO FE>90 (V vs. RHE)	Ref
Disordered Ag	0.1 M $KHCO_3$	96~90	-0.6~-1.7	This work
Triangular Ag nanoplates	0.1 M $KHCO_3$	97~90	-0.75~-0.95	17
Cu/SnO_2 NPs	0.5 M $KHCO_3$	93~90	-0.58~-0.9	S4
Pd icosahedra	0.1 M $KHCO_3$	90	-0.7~-0.9	S5
OD Ag	0.1 M $KHCO_3$	95~90	-0.8~-1.0	S6
Zn hierarchical	0.5 M KCl	92~90	-0.8~-1.1	15
Pd NPs	0.1 M $KHCO_3$	91~90	-0.7~-1.0	16
Au concave rhombic dodecahedra	0.5 M $KHCO_3$	93~90	-0.47~-0.7	S7
Ag Nanocoral	0.1 M $KHCO_3$	92~90	-0.45~-0.7	S8
OD-Au	0.5 M $NaHCO_3$	94~90	-0.45~-0.7	S9
Au Nanowire	0.5 M $KHCO_3$	94~90	-0.33~-0.55	14
Au NPs	0.5 M $KHCO_3$	97~90	-0.4~-0.7	S10

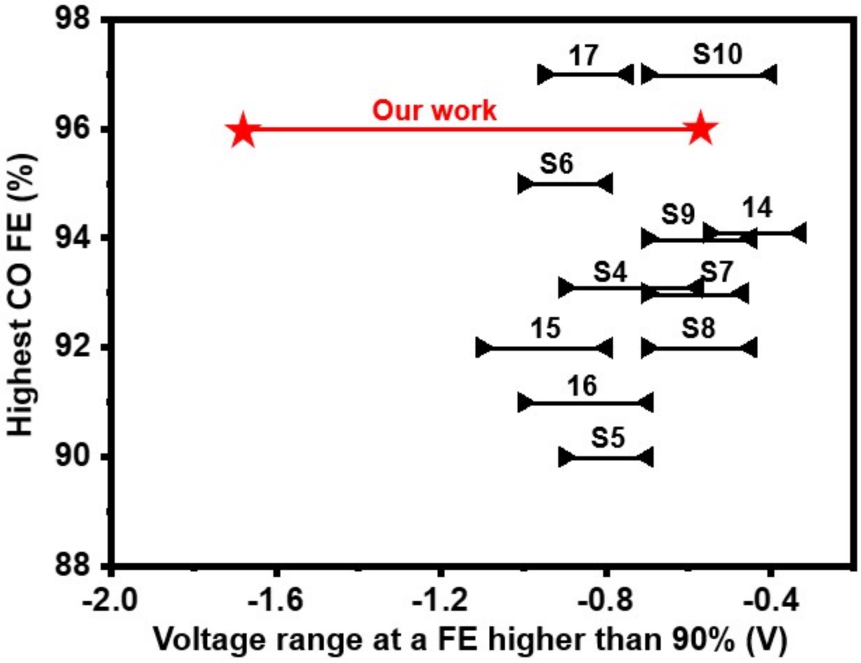

Figure S6. Comparison of different metal electrocatalysts for CO_2 reduction to CO.

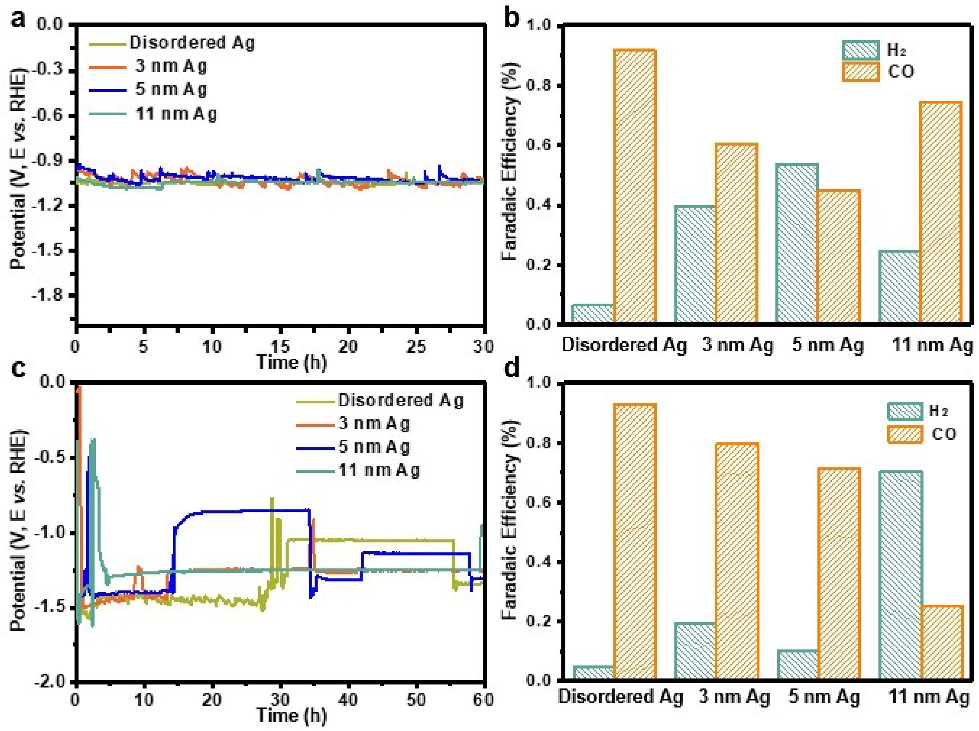

Figure S7. (a, c) Cathode voltage in PV-EC system driven by a ten-section a-Si solar cell which is constructed by concatenating a six-section a-Si solar cell with another one that obscures 1/3 areas and respectively. (b, d) CO Faradaic efficiency in PV-EC system driven by a ten-section a-Si solar cell and random sheltered a twelve-section a-Si solar cell, respectively.

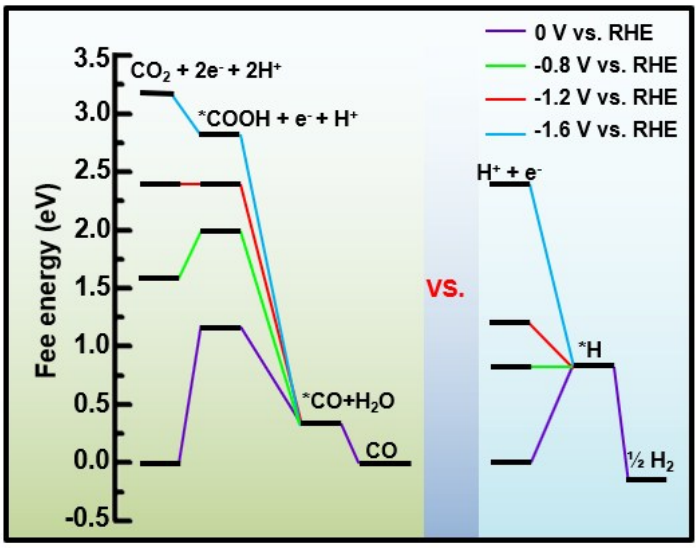

Figure S8. Free energy diagram for CO_2RR and HER at different potential on Ag surface.[17, 23]

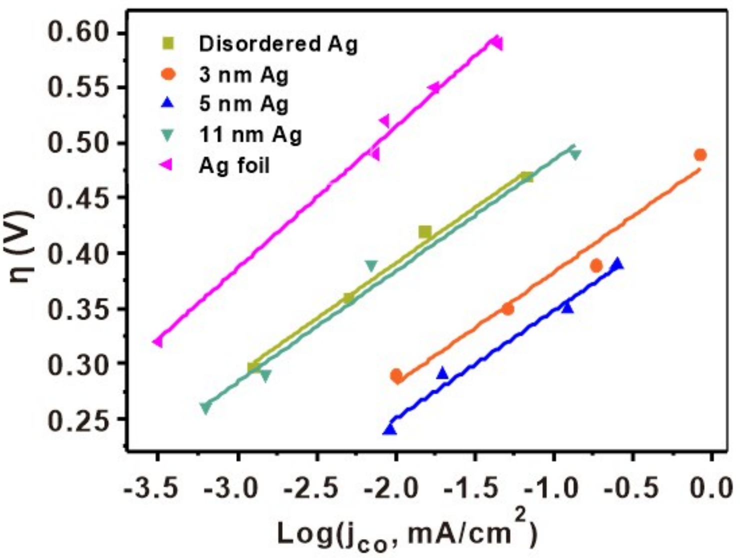

Figure S9. CO Tafel plots of disordered, 3 nm, 5 nm, 11 nm Ag and Ag foil.

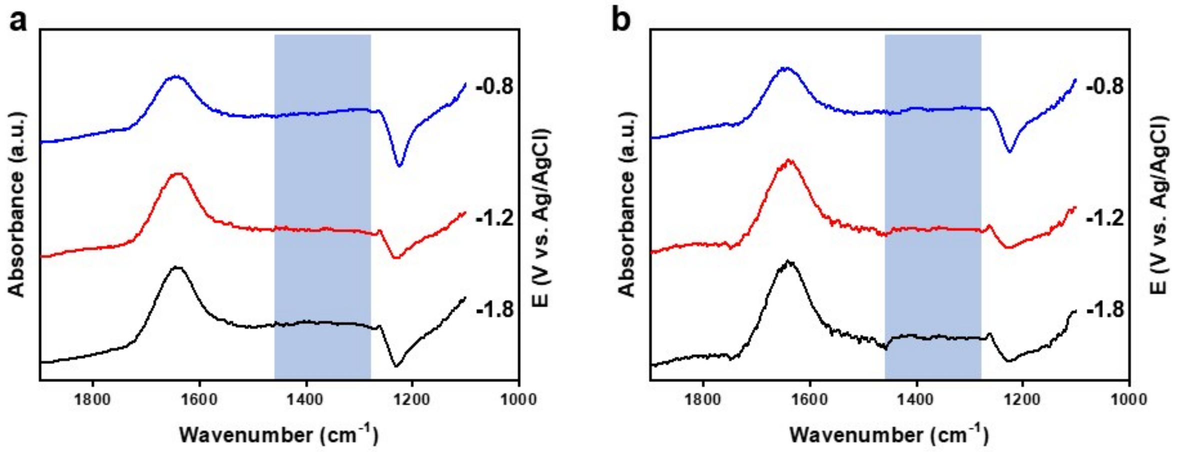

Figure S10. ATR-SEIRAS spectra of (a) Au in CO_2 saturated 0.1 M KCl electrolyte and (b) disordered Ag-C on Au in Ar saturated 0.1 KCl electrolyte.

Table S2. Band Assignments in Figure 4a.

Wavenumber (cm^{-1})	Assignment
1650	δ(H-O-H) of H_2O[S11]/ν(C=O) asymmetric stretching of *COOH[25-28]
1450	Solution CO_3^{2-} [S12]
1378	symmetric *COO- stretching and C-O stretching of *COOH[28]
1290	C-OH stretch of *COOH[25, 26]
1210	Si-O-Si[S13-S14]

References

[S1] H. Miyake, S. Ye, M. Osawa, *Electrochem. Commun.* **2002**, 4, 973-977.

[S2] L. Nagahara, T. Ohmori, K. Hashimoto, A. Fujishima, *J. Vac. SCI. Technol.* **1993**, 11, 763-767.

[S3] M. Dunwell, Q. Lu, J. M. Heyes, J. Rosen, J. G. Chen, Y. Yan, F. Jiao, B. Xu, *J. Am. Chem. Soc.* **2017**, 139, 3774-3783.

[S4] Q. Li, J. Fu, W. Zhu, Z. Chen, B. Shen, L. Wu, Z. Xi, T. Wang, G. Lu, J.-j. Zhu, *J. Am. Chem. Soc.* **2017**, 139, 4290-4293.

[S5] H. Huang, H. Jia, Z. Liu, P. Gao, J. Zhao, Z. Luo, J. Yang, J. Zeng, *Angew. Chem. Int. Ed.* **2017**, 56, 3594-3598.

[S6] M. Ma, B. J. Trzesniewski, J. Xie, W. A. Smith, *Angew. Chem. Int. Ed*. **2016**, 55, 9748-9752.

[S7] H. E. Lee, K. D. Yang, S. M. Yoon, H. Y. Ahn, Y. Y. Lee, H. Chang, D. H. Jeong, Y.-S. Lee, M. Y. Kim, K. T. Nam, *ACS nano* **2015**, 9, 8384-8393.

[S8] Y.-C. Hsieh, S. D. Senanayake, Y. Zhang, W. Xu, D. E. Polyansky, *ACS Catal.* **2015**, 5, 5349-5356.

[S9] X. Feng, K. Jiang, S. Fan, M. W. Kanan, *J. Am. Chem. Soc*. **2015**, 137, 4606-4609.

[S10] W. Zhu, R. Michalsky, O. Metin, H. Lv, S. Guo, C. J. Wright, X. Sun, A. A. Peterson, S. Sun, *J. Am. Chem. Soc*. **2013**, 135, 16833-16836.

[S11] K. I. Ataka, T. Yotsuyanagi, M. Osawa, *J. Phys. Chem. C* **1996**, 100, 10664-10672.

[S12] Z. H. Cheng, A. Yasukawa, K. Kandori, T. Ishikawa, *J. Chem. Soc. Faraday Trans.* **1998**, 94, 1501-1505.

[S13] H. Ou-Yang, E. P. Paschalis, W. E. Mayo, A. L. Boskey, R. Mendelsohn, *J. Bone Miner. Res.* **2001**, 16, 893–900.

[S14] R. Tian, O. Seitz, M. Li, W. W. Hu, Y. J. Chabal, J. Gao, *Langmuir* **2010**, 26, 4563-4566.
